# Unveiling the paradigm shift: systemic treatment strategies in small, node-negative breast cancer

**DOI:** 10.1038/s41523-025-00761-8

**Published:** 2025-05-16

**Authors:** Mariana Carvalho Gouveia, Mariana Scaranti, Bruna Migliavacca Zucchetti, Renata Colombo Bonadio, Romualdo Barroso-Sousa, Jose Pablo Leone

**Affiliations:** 1 Department of Medical Oncology, DASA Oncology, 9 de Julho Hospital, São Paulo, Brazil; 2 Department of Medical Oncology, Instituto D’Or de Pesquisa e Ensino (IDOR), São Paulo, Brazil; 3 Department of Medical Oncology, DASA Oncology, Brasília Hospital, DASA, Brasília, Brazil; 4 Department of Medical Oncology, Dana-Farber Cancer Institute, Harvard Medical School, Boston, USA

**Keywords:** Cancer, Oncology, Breast cancer, Cancer therapy

## Abstract

The implementation of screening mammography has significantly altered the size distribution of breast tumors, with approximately 20% of newly diagnosed breast cancers measuring 10 mm or smaller with node-negative disease (T1aN0 and T1bN0). The management of these early-stage breast cancers remains a subject of debate. Historically, patients with T1aN0 and T1bN0 breast cancer have been excluded from adjuvant chemotherapy trials due to their excellent prognosis, with reported 10-year disease-specific survival rates exceeding 90%. However, the optimal treatment strategy for this subgroup of patients continues to be controversial, as the potential benefits of adjuvant chemotherapy must be carefully weighed against the risks of overtreatment. In this review, we summarize current evidence on outcomes and treatment strategies, highlight gaps in the literature, and provide future perspectives on the management of T1aN0 and T1bN0 breast cancer, according to immunohistochemical subtypes.

## Introduction

The widespread adoption of mammography as a screening tool has led to a notable rise in the detection of early-stage breast cancer over recent decades. This has caused a shift in the size distribution of breast tumors, with a significant increase in the detection of small breast lesions. Between 1975 and 2010, the proportion of invasive tumors measuring less than 1.0 cm increased from 6% to 18%^1,2^.

Patients diagnosed with localized tumors categorized as T1a (invasive primary cancer measuring 0.1-0.5 cm) and T1b (invasive primary cancer measuring 0.6-1.0 cm) node-negative tumors have excellent outcomes, with a 5-year disease-free survival (DFS) exceeding 90% and a 10-year probability of breast cancer specific mortality of only 4%^3–5^. While tumor size plays a critical role in prognostication in T1a and T1b tumors, immunohistochemical subtype is another significant factor^6–10^.

Patients with node-negative tumors measuring 1.0 cm or smaller were systematically excluded from major randomized adjuvant chemotherapy trials. Consequently, the benefit of chemotherapy in this subgroup has not been confirmed. In other words, when a patient with these tumor characteristics asks about the absolute benefit of adjuvant chemotherapy compared to no chemotherapy at all, we lack a definitive answer. The paucity of randomized prospective data explains the absence of a clear consensus on the benefits of this treatment for T1aN0 and T1bN0 disease^11,12^.

The aim of this review is to explore the paradigm shift in treatment strategies and to highlight the outcomes of small, node-negative T1aN0M0 and T1bN0M0 tumors based on their immunohistochemical subtype.

## Hormone receptor-positive/HER2-negative breast cancer

Hormone receptor (HR)-positive/HER2-negative breast cancer represents 60 to 75% of all cases and generally has a favorable prognosis compared to other subtypes^13^. Among HR-positive/HER2-negative breast cancer, 5-year overall survival (OS) and breast cancer-specific survival exceed 95% in T1a and T1b tumors, and invasive disease-free survival (IDFS) and distant relapse-free survival (DRFS) exceed 90%, regardless of adjuvant chemotherapy. Therefore, the mainstay of systemic treatment for these patients is endocrine therapy. In a retrospective analysis, patients with higher-grade tumors who were not treated with chemotherapy (*N* = 434) had numerically lower DRFS (94%) compared to those who received (*N* = 92) adjuvant chemotherapy (98%), without statistical significance^14^. However, no randomized data exist to confirm this.

Meta-analyses of randomized clinical trials have evaluated the benefit of adjuvant chemotherapy in early-stage breast cancer^15,16^. In these pooled analyses, patients who received adjuvant chemotherapy showed a reduction in the risk of recurrence, with a relative benefit irrespective of recorded patient and tumor characteristics, including age, hormone receptor status, tumor size, tumor grade, histological type, and nodal status. However, the absolute benefit depends on the risk of recurrence. For some patients, the absolute benefits may not justify the short-term toxicity and longer-term risks of cardiovascular disease and leukemia. The largest meta-analysis evaluating chemotherapy benefit excluded T1a and T1b node-negative tumors, and given their prognosis, chemotherapy benefit seems unlikely^15,16^ Unfortunately, no prospective trial has specifically evaluated this population.

Nowadays, several gene-based assays have illuminated the genomic characteristics of HR-positive/HER2-negative breast cancer and help to guide decisions regarding adjuvant chemotherapy^17^. The 21-gene Oncotype DX testing is a reliable and widely used one. The TAILOR-X trial, a prospective, randomized study, evaluated HR-positive/HER2-negative, node-negative breast cancer with intermediate recurrence score (RS) 11–25 in tumors measuring 1.1 to 5.0 cm (or 0.5 to 1.0 cm with unfavorable histological features, defined as an intermediate or poor nuclear and/or histologic grade 2 or 3, or lymphovascular invasion). Patients were randomly assigned to endocrine therapy versus chemoendocrine therapy. The primary endpoint was IDFS, and the trial was designed to demonstrate the non-inferiority of endocrine therapy alone. The study showed that adjuvant endocrine therapy was not inferior to chemoendocrine therapy in the intention-to-treat analysis. However, it is important to note that only T1b patients with unfavorable histological features were included in the trial. Those with favorable characteristics likely do not require a genomic assay to avoid chemotherapy. Moreover, T1b patients were underrepresented, comprising only 13% of the study population (869 patients)^18^.

Another genomic assay, the MammaPrint 70-gene signature, can help identify patients who benefit from adjuvant chemotherapy. This assay was evaluated in the MINDACT trial, a randomized, phase III study that assessed early-stage breast cancer (T1, T2, or operable T3) with up to three positive lymph nodes. Patients with discordant clinical and genomic risk were randomly assigned to receive chemotherapy or not. The primary endpoint tested whether the five-year distant metastasis-free survival rate in patients with high clinical risk and low genomic risk not receiving chemotherapy was above a predefined non-inferiority boundary of 92%. The study demonstrated that the 70-gene signature could safely guide the omission of chemotherapy in patients with high clinical risk but low genomic risk, with only a 1.5% increase in the risk of distant metastasis at five years^19^. An exploratory analysis of the MINDACT, evaluating T1aN0 and T1bN0 breast cancer patients, was recently published. Of 6693 patients enrolled, 715 were node-negative HR-positive/HER2-negative small tumors (T1a n = 34, T1b n = 681). Among them, all were classified as clinical low-risk, with 17.3% categorized as genomic high-risk by the MammaPrint assay. For genomic high-risk tumors, 8-year distant metastasis-free survival (DMFS) was 89.2% (95% CI 73.6–95.8%) among those who received chemotherapy and 94.1% (95% CI 82.9–98.1%) without chemotherapy, respectively. Despite the small number of randomized patients, genomic high-risk breast tumors did not seem to derive benefit from chemotherapy^20^.

A retrospective study of 237 patients with T1bN0 disease examined adjuvant chemotherapy decisions in a multidisciplinary team setting. Initial decisions were made using clinicopathological findings without 21-gene recurrence scores, followed by final recommendations after incorporating recurrence scores. Adjuvant chemotherapy was recommended for 31.6% of patients, particularly those with higher tumor grade (odds ratio [OR] = 2.99 for grade II; OR = 59.19 for grade III, *P* = 0.006), lymphovascular invasion (OR = 8.22, P = 0.032), Luminal-B subtype (OR = 5.68, *P* < 0.001), and intermediate to high-risk recurrence scores (OR = 10.01 for intermediate-risk, OR = 192.42 for high-risk, P < 0.001). Treatment plans changed for 18.6% of patients after recurrence scores were revealed, with 42 patients switching to chemotherapy and only two switching away. Multivariate analysis found that estrogen receptor expression (P = 0.011), progesterone receptor expression (P < 0.001), and Ki-67 index (P = 0.001) were independently associated with recurrence score distribution^21^.

In summary, T1a patients were not eligible for the adjuvant chemotherapy trials or TAILOR-X. Thus, we have no prospective evidence to recommend chemotherapy for these patients. For T1b patients, no prospective randomized data on the benefit of adjuvant chemotherapy exist. However, they were eligible for TAILOR-X if they had grade 2 or 3 tumors or lymphovascular invasion. Therefore, Oncotype DX may be performed in these patients to confirm the safety of omitting chemotherapy for those with RS up to 25.

## HER2positive breast cancer

It is well established that, in general, HER2 amplification is a predictor of poor breast cancer outcomes^8^. Considering different trials in this setting, there is less than a 10% risk of recurrence at five or more years for patients with T1aN0 and T1bN0 HER2-positive breast cancers^22^. However, small, node-negative cancers were not eligible for inclusion in the adjuvant randomized trastuzumab trials. Because of this, it is uncertain whether chemotherapy with trastuzumab improves outcomes in this population^23–27^.

A challenge in clinical practice is that we do not know the outcomes for patients with tumors smaller than 1 cm if left untreated. In fact, recent data suggest that patients with T1a or T1b tumors that are HER2-positive may fare equally well without treatment^28^.

A meta-analysis evaluated patients with a primary tumor size less than or equal to 2 cm regardless of lymph node status in the randomized adjuvant trastuzumab trials. Only 75 patients had T1a or T1b disease, reflecting the paucity of these patients in the original adjuvant trials. The meta-analysis does not disclose the outcomes for these patients. In fact, the authors acknowledge that almost all patients included in the meta-analysis had T1c tumors and that there is no definitive evidence available from the 5 adjuvant trastuzumab trials with regards to the benefit of adjuvant trastuzumab in patients with T1a and T1b tumors^29^.

In this context, the APT trial was designed as a single-arm phase II study that enrolled 410 patients with node-negative disease measuring up to 3 cm (31% with T1b lesions and 17% with T1a lesions) to receive paclitaxel weekly for 12 weeks plus trastuzumab for one year (TH regimen). Outcomes were satisfactory, with seven-year and ten-year DFS of 93% and 91%, and OS rates of 95% and 94%, respectively. Overall toxicity showed 13 patients (3%) reporting at least one episode of grade 3 neuropathy, two patients reporting grade 3 left ventricular systolic dysfunction, and 13 patients requiring trastuzumab discontinuation due to a significant asymptomatic decline in the ejection fraction^30,31^.

The efficacy and safety of adjuvant T-DM1 in this setting were explored in the ATEMPT trial. This randomized phase II trial included 497 stage I HER2-positive breast cancer patients (approximately half with tumors larger than 1 cm), assigned in a 3:1 ratio to 1 year of T-DM1 versus TH regimen. At a median follow-up of 5.8 years, 11 IDFS events were observed in the T-DM1 arm, with a five-year IDFS of 97.0% (95% CI, 95.2–98.7), compared to nine events in the TH arm, with a five-year invasive IDFS of 91.1% (95% CI, 85.7–96.8). It is important to note that the study was not powered to detect efficacy differences between the two regimens^32,33^. (Table 1).

**Table 1:** Comparison between the main trials for small, node-negative HER2-positive breast tumors

Comparison between APT trial and ATEMPT		
	**APT Trial**^30^	**ATEMPT trial**^32^
**Phase**	II	II
**N**	410	497
**Eligibility**	Small ( ≤ 3 cm), node-negative, HER2-positive breast cancer	Small ( ≤ 2 cm), node-negative, HER2-positive breast cancer
**Regimen**	TH regimen [T 80 mg/m^2^ IV with H once every week × 12 weeks (4 mg/kg load →2 mg/kg), followed by H × 39 weeks (6 mg/kg once every 3 weeks)]	T-DM1 3.6 mg/kg IV every 3 weeks for 17 cycles or TH [T 80 mg/m^2^ IV with H once every week × 12 weeks (4 mg/kg load →2 mg/kg), followed by H × 39 weeks (6 mg/kg once every 3 weeks)]
**Primary endpoint**	3-year iDFS	Incidence of clinically relevant toxicities with T-DM1 versus TH and to evaluate iDFS for T-DM1
**Tumor size**	T1a: 68 patients T1b: 124 patients	T1a: 70 patients T1b: 167 patients
**3-year iDFS**	98.7%(95% CI, 97.6 to 99.8).	T-DM1 arm: 97.8%(95% CI, 96.3 to 99.3) TH arm: 93.4%(95% CI, 88.7 to 98.2)

*HER2* Human Epidermal growth factor Receptor-type 2, *N* Number of patients, *T* Paclitaxel, *H* Herceptin, *T-DM1* Trastuzumab Emtansine, *iDFS* Invasive disease free-survival, *CI* Confidence interval, *IV* intravenous.

When considering adverse events between T-DM1 and TH, clinically relevant toxicities were similar: 46% in the T-DM1 arm and 47% in the TH arm (P = 0.83). However, discontinuations of all protocol therapy were 18% and 6% for T-DM1 and TH, respectively. The most common toxicities for T-DM1 discontinuation were elevated liver enzymes or bilirubin (28%), neuropathy (19%), and thrombocytopenia (19%). On the other hand, in 18-month global health-related quality of life T-DM1 was associated with better physical well-being, less activity impairment and lower odds of neuropathy (11 versus 23%, *p* = 0.0031)^34^. In a scenario where treatment efficacy and prognosis were sustained, future data on the efficacy, adverse events and quality of life of a shorter course of T-DM1 may reshape recommendations for T-DM1 in the adjuvant setting.

Based on these studies, current guidelines recommend considering or offering adjuvant treatment for node-negative small tumors. Despite the results of the ATEMPT trial, guidelines still support the use of the TH regimen for T1N0 tumors^11,35^.

Two ongoing trials offer future perspectives for stage I HER2-positive breast cancer: ATEMPT 2.0 (NCT04893109), evaluating a shorter exposure to T-DM1 regimen (six cycles), and the phase II ADEPT trial (NCT04569747), a phase II study exploring a chemotherapy-free regimen combining endocrine therapy with subcutaneous trastuzumab and pertuzumab for patients with HER2-positive breast cancer expressing hormone receptors. These trials are significant as they evaluate treatment de-escalation for HER2-positive breast cancer, including tumors larger than 1 cm (Table 2).

**Table 2:** Future perspectives on de-escalation for small, node-negative HER2-positive tumors

Perspectives for small, node-negative HER2-positive breast tumors		
	**ATEMPT 2.0**	**ADEPT**
**Phase**	Randomized, phase II	Single-arm, phase II
**Estimated N**	500	375
**Eligibility**	Small ( ≤ 2 cm), node-negative, HER2-positive breast cancer	Small ( ≤ 2 cm), node-negative, HR-positive, HER2-positive breast cancer
**Arm 1**	T-DM1 IV every 3 weeks for 6 cycles, followed by Trastuzumab SC every 3 weeks for 11 cycles	Trastuzumab + Pertuzumab SC Every 3 weeks for 18 cycles and hormonal therapy for 5 years
**Arm 2**	Paclitaxel for 12 weeks (4 cycles) and Trastuzumab SC every 3 weeks for 17 cycles.	Single-arm
**Primary endpoint**	iDFS in the T-DM1 followed by trastuzumab SC arm up to 72 months and incidence of clinically relevant toxicities in both arms	iDFS at 3 years

*HER2* Human Epidermal growth factor Receptor-type 2, *HR* Hormone receptor, *N* Number of patients, *T-DM1* Trastuzumab Emtansine, *iDFS* Invasive disease free-survival, *IV* intravenous, *SC* Subcutaneous.

Nevertheless, despite the results presented suggesting favorable outcomes of de-escalated therapies in stage I HER2-positive breast cancer, no trials have confirmed that tumors smaller than 1 cm benefit from chemotherapy with trastuzumab compared to no systemic treatment. Many patients with these subcentimeter tumors may have excellent outcomes without any adjuvant systemic therapy, and the challenge remains in identifying those at higher risk.

In the era of precision medicine, tailoring adjuvant treatment to the patient’s tumor is the goal. In this regard, comprehensive tumor biology assessment using the HER2DX tool could identify patients at significantly higher risk of recurrence, as demonstrated in the APT (4.9%) and ATEMPT (6.4%) trials. This underscores the promise of HER2DX in tailoring adjuvant treatment for small HER2-positive, node-negative tumors, warranting further prospective validation^31,33^.

## Triple-negative breast cancer

Stage I triple-negative breast cancer accounts for one-third of all triple-negative breast cancers and almost one-third of these are T1a and T1b tumors. For patients with triple-negative breast cancer who were untreated with chemotherapy, the 5-year disease recurrence-free survival for T1a tumors was 93% (95% CI, 84% to 97%; *n* = 74) and for T1b tumors was 90% (95% CI, 81% to 95%; *n* = 94)^14^.

A population-based retrospective study using Surveillance, Epidemiology, and End Results (SEER) database evaluated adjuvant chemotherapy treatment patterns and survival outcomes among patients diagnosed with stage IA triple-negative breast cancer between 2010 and 2019. The study included 8,601 patients, of whom 11% had T1a tumors and 25% had T1b tumors. Chemotherapy use among T1a tumors did not change significantly over time ( < 30% across all years) but increased significantly among T1b tumors (*p* = 0.001), reaching ≥60% of patients in this stage. For breast cancer-specific survival, T1a tumors had favorable outcomes regardless of chemotherapy, with only 17 (1.8%) breast cancer deaths. For T1b cancers, no significant association between chemotherapy and breast cancer specific-survival was observed, with 63 (2.9%) breast-cancer deaths^36^. This study highlights the risk of overtreatment and unnecessary toxicity, as these patients have excellent outcomes despite chemotherapy, with no correlation between adjuvant chemotherapy and breast cancer-specific survival, even for T1b tumors^37,38^.

In a scenario where the absolute benefit of chemotherapy for T1a and, to a lesser extent, T1b triple-negative breast cancer is small or absent, biomarkers are needed. A cohort study evaluated 1,041 stage I triple-negative breast cancer untreated with chemotherapy according to tumor-infiltrating lymphocytes (TILs). In the overall cohort, TILs levels of at least 30% were associated with better breast cancer-specific survival compared with TILs levels less than 30% (96% vs. 87%; HR 0.45; 95% CI, 0.26-0.77). However, the role of TILs levels in T1a and T1b tumors is less clear, as no correlation between TILs levels and breast cancer-specific survival was observed in this subgroup. The excellent outcomes of patients with pT1a and T1b tumors, combined with low event rates, suggest that larger cohorts and longer follow-up are needed to define the role of TILs levels in T1a and T1b triple-negative breast cancer. On the other hand, for T1c tumors, TILs levels ≥ 30% versus <30% (HR 0.24; 95% CI, 0.10–0.60) and ≥50% versus <50% (HR 0.27; 95% CI, 0.10–0.74) were associated with improved outcomes, with an excellent 10-year breast cancer-specific survival of 95%^39^.

Additionally, survival outcomes for patients with T1bN0 triple-negative breast cancer differed according to TILs levels in another study. TILs levels ≥50% identified patients with 5-year recurrence-free survival, DRFS, and OS rates approaching or exceeding 90%, in patients without chemotherapy^40^. Another study selected 441 chemotherapy naïve, young patients ( < 40 years) with node-negative (almost 40% T2 tumors) triple-negative breast cancer from a Dutch population-based registry. In their analysis, patients with high TILs ( ≥ 75%: 21%) had excellent prognosis, with a 15-year cumulative incidence of distant metastasis or death of only 2.1% (95% CI 0–5.0). Moreover, every 10% increment of TILs reduced the risk of death by 19% (adjusted HR 0.81, 95% CI 0.76–0.87). These intriguing findings suggest that current multiagent chemotherapy regimens may have a limited additional benefit in reducing recurrence and mortality in this population with high expression of TILs because even in young patients and bigger tumors the excellent prognosis was sustained^41^. However, further studies are needed (Table 3).

**Table 3:** Tumor infiltrating lymphocytes for triple negative breast cancer

	Geurts et al. ^39^	Leon-Ferre et al. ^40^	De Jong et al. ^41^
**N**	1041	1966	441
**Eligibility**	Stage I TNBC	Early-stage TNBC (Stage I-III)	Node-negative TNBC with < 40 years
**Chemo**	No	No	No
**TILs**	<30%: 74.4% 30–74%: 12% ≥ 75%: 13.5%	≥30%: 35% ≥50%: 21%	<30%: 52% 30–74%: 27% ≥ 75%: 21%
**T1abN0**	T1aN0: 8.6% T1bN0: 38.7%	T1aN0: 5.6% T1bN0: 10.8%	T1aN0: 0.5% T1bN0: 7.2%
**Outcomes**	Overall, T1ab tumors had good outcomes [10-year BCSS was 92% (95% CI, 89%-94%)], and TILs at the prespecified cutoffs were not associated with BCSS in this subset	Every 10% increment of TILs decreased the risk of death by 12% (HR 0.88, 95% CI, 0.85-0.91)	Every 10% increment of TILs decreased the risk of death by 19% (adjusted HR 0.81, 95% CI, 0.76 to 0.87)

*N* Number of patients, Chemo Chemotherapy, *TNBC* Triple-negative breast cancer, *TILs* tumor infiltrating lymphocytes, *HR* Hazard ratio, *CI* confidence interval, *BCSS* Breast cancer specific survival.

Looking to the future, ongoing trials aim to validate the de-escalation of treatment for small node-negative tumors. The OPTIMAL trial (NCT06476119) is an international, open-label, patient-preference study enrolling patients with stage I triple-negative breast cancer and a high TILs score (defined as ≥ 50% for patients ≥ 40 years; ≥ 75% for patients < 40 years), which will assess whether adjuvant chemotherapy can be safely omitted.

Another perspective beyond TILs is the use of novel genomic tools. As an example, TNBC-DX is a test that evaluates 10-gene Core Immune Gene module, the 4-gene tumor cell proliferation signature, tumor size, and nodal staging. In a validation cohort, in early-stage triple-negative breast cancer treated with neoadjuvant taxane-based chemotherapy, TNBC-DX scores were significantly associated with pathological complete response, event-free-survival and OS^42^. Potentially, this comprehensive risk assessment could be a useful tool to distinguish those small tumors that benefit from adjuvant chemotherapy in a clinical setting.

Currently, the National Comprehensive Cancer Network guidelines recommend considering adjuvant chemotherapy for tumors measuring 0.6–1 cm (T1b). Adjuvant chemotherapy is not recommended for tumors ≤0.5 cm (T1a), but in selected high-risk cases—such as young patients with high-grade histology—adjuvant chemotherapy may be considered^35^. Conversely, the European guidelines recommend adjuvant chemotherapy for T1b tumors while suggesting it may not be necessary for T1a tumors^11^.

## Conclusion

While the prognosis of T1a and T1b node-negative breast tumors is generally favorable, a minority of patients may eventually experience recurrence, and we do not know who those patients are. Currently, there remains a significant lack of prospective data to support the benefits of adjuvant chemotherapy in this population. Clinical practice often leans towards prescribing such treatments driven by patient or physician anxiety about recurrence and the pervasive but mistaken belief that more aggressive treatment is always better. However, it is imperative to approach treatment decisions thoughtfully, carefully weighing potential benefits against the risks of side effects and the impact on overall quality of life. In some cases, the small absolute benefit of certain treatments may not justify their associated toxicities. In other cases, patients may experience recurrence despite undergoing adjuvant chemotherapy. As such, a cautious approach is essential (Table 4).

**Table 4:** NCCN and ESMO recommendations on adjuvant treatment in T1a and T1b node-negative breast cancer tumors

	HER2-positive	HR-positive/HER2-negative	Triple-negative
**NCCN guidelines**^35^			
**T1a N0**	Consider adjuvant chemotherapy with trastuzumab.^a, b, c^	No adjuvant chemotherapy. Consider adjuvant endocrine therapy.	No adjuvant chemotherapy.
**T1b N0**	Consider adjuvant chemotherapy with trastuzumab.^a, b, c^	Adjuvant chemotherapy followed by endocrine therapy ± ovarian suppression. Consider adjuvant ribociclib for eligible patients.	Consider adjuvant chemotherapy.^d^
**ESMO guidelines**^11^			
**T1a N0**	Adjuvant chemotherapy with trastuzumab.	No adjuvant chemotherapy. Endocrine therapy is indicated.	Consider no adjuvant chemotherapy.
**T1b N0**	Adjuvant chemotherapy with trastuzumab.	No adjuvant chemotherapy. Endocrine therapy is indicated.	4–8 cycles of systemic chemotherapy.

^a^This is a population of patients with breast cancer that was not studied in the available randomized trials. The decision for use of trastuzumab therapy in this cohort of patients must balance the known toxicities of trastuzumab, such as cardiac toxicity, and the uncertain, absolute benefits that may exist with trastuzumab therapy.

^b^Adjuvant chemotherapy with weekly paclitaxel and trastuzumab can be considered for pT1N0M0, HER2-positive cancers, particularly if the primary cancer is HR-negative.

^c^ If HR-positive disease, consider endocrine therapy for T1aN0M0 and endocrine therapy is recommended for T1bN0M0.

^d^In selected patients with high-risk features (for example, young patients with high-grade histology), adjuvant chemotherapy may be considered (category 2B).

*NCCN* National Comprehensive Cancer Network, *ESMO* European Society for Medical Oncology, *HER2* Human Epidermal growth factor Receptor-type 2, *HR* Hormone receptor.

In the context of subcentimeter breast tumors, a careful selection process for adjuvant systemic therapy is essential. Widespread use of chemotherapy could unnecessarily expose many patients to toxic treatments while benefiting only a small subset. Beyond clinical and pathological features, genomic assays may help refine this selection. Dedicated trials for this patient population are crucial to generating robust evidence, developing tailored treatment strategies, and integrating molecular tools to improve patient selection and optimize outcomes.

## Data Availability

No datasets were generated or analysed during the current study.
